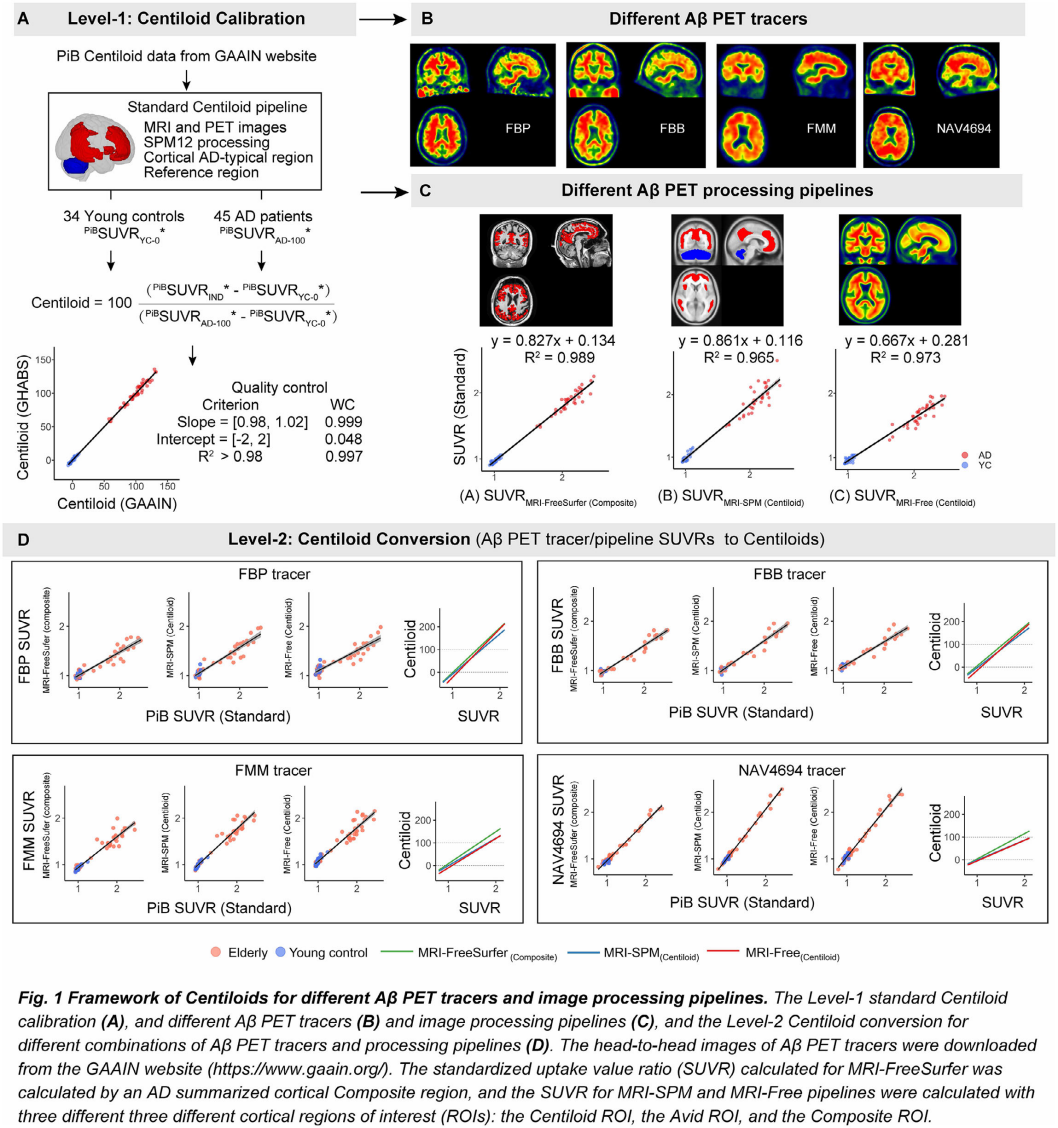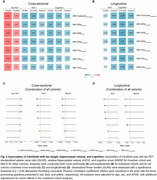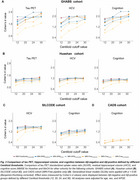# Comparisons of Aβ Centiloids with Different Pipelines in Alzheimer's Disease Across Multicenter Chinese Aging Cohorts

**DOI:** 10.1002/alz70856_101806

**Published:** 2025-12-25

**Authors:** Pan Sun, Xin Zhou, Ying Han, Yan‐Jiang Wang, Fang Xie, Tengfei Guo

**Affiliations:** ^1^ Shenzhen Bay Laboratory, Shenzhen, Guangdong, China; ^2^ Hainan University, Haikou, Hainan, China; ^3^ Xuanwu Hospital of Capital Medical University, Beijing, Beijing, China; ^4^ Daping Hospital, Third Military Medical University, Chongqing, China; ^5^ Huashan Hospital, Fudan University, Shanghai, Shanghai, China; ^6^ Institute of Biomedical Engineering, Shenzhen Bay Laboratory, Shenzhen, China; ^7^ Peking University Shenzhen Graduate School, Shenzhen, Guangdong, China

## Abstract

**Background:**

The variability of β‐amyloid (Aβ) tracers, processing pipelines, and thresholds in Aβ positron emission tomography (PET) imaging raises the importance of establishing Centiloid to assess cortical Aβ plaques, particularly in Chinese Alzheimer's disease (AD) research and clinical trials.

**Method:**

We standardized the conversions to Centiloid units across three processing pipelines: (1) the MRI‐FreeSurfer pipeline, with an AD summarized cortical region as the standardized uptake value ratio (SUVR) in a Composite region (SUVR_MRI‐FreeSurfer (Composite)_), (2) the MRI‐SPM pipeline, with three different cortical ROIs (SUVR_MRI‐SPM (Centiloid)_, SUVR_MRI‐SPM (Avid)_, and SUVR_MRI‐SPM (Composite)_, and (3) the MRI‐Free pipeline, also with the three different cortical ROIs above. The Centiloids calculation coefficients were determined for ^18^F‐NAV4694, ^18^F‐Florbetaben (FBB), ^18^F‐florbetapir (FBP), and ^18^F‐flutemetamol (FMM) using these pipelines above. To evaluate the performance of the Centiloids in detecting AD‐related pathologies, we calculated the Centiloids for FBP across four representative Chinese aging cohorts (Southern China: the Greater‐Bay‐Area Healthy Aging Brain Study (GHABS, *n* = 112); Northern China: the Sino Longitudinal Study on Cognitive Decline (SILCODE, *n* = 108); Eastern China: Huashan Hospital cohort (*n* = 777); and Western China: the Chongqing Ageing & Dementia Study (CADS, *n* = 182). We subsequently analyzed the association of the Centiloids with tau PET, residual hippocampal volume (rHCV), and cognition. Further, we investigated the difference in tau, rHCV, and cognition between Aβ‐negative (A−) and Aβ‐positive (A+) individuals defined by different Centiloid thresholds in Chinese aging populations.

**Result:**

The MRI‐FreeSurfer and MRI‐SPM pipelines provided the most consistent results, with the MRI‐Free pipeline also yielding reliable conversion to Centiloid units for four Aβ PET tracers (Figure 1). Higher Centiloids were associated with more tau PET SUVR, more decreases or faster decline rates in rHCV and cognition (Figure 2). Additionally, different thresholds of Centiloid generated distinct effect size in tau PET SUVR, rHCV, and cognitive score between A− and A+ groups (Figure 3).

**Conclusion:**

This multiple Aβ PET study established the standard calculation coefficients of Centiloid in different settings in the Chinese aging population. Our findings provide significant reference and support for Aβ Centiloids harmonization in AD studies and clinical trials in China.